# Removal capability, implant-abutment connection damage and thermal effect using ultrasonic and drilling techniques for the extraction of fractured abutment screws: an in vitro study

**DOI:** 10.1186/s12903-022-02653-w

**Published:** 2022-12-14

**Authors:** María Bufalá Pérez, Álvaro Zubizarreta-Macho, Javier Borrajo Sánchez, Jorge Hernández Rodríguez, Jorge Alonso Pérez-Barquero, Elena Riad Deglow, Sofía Hernández Montero

**Affiliations:** 1 Department of Implant Surgery, Faculty of Health Sciences, Alfonso X El Sabio University, Avda. Universidad, 1., 28691 Villanueva de la Cañada, Madrid Spain; 2 Department of Surgery, University of Salamanca, Salamanca, Spain; 3 Department of Biomedical and Diagnostic Sciences, University of Salamanca, Salamanca, Spain; 4 Department of Stomatology, Faculty of Medicine and Dentistry, University of Valencia, 46010 Valencia, Spain

**Keywords:** Dental implants, Fractured, Screw-retained, Abutment screw, Dental prostheses, Implant-supported protheses

## Abstract

The aim of this work was to analyze and compare the removal capability, conical internal hex implant-abutment connection damage and thermal effect using ultrasonic and drilling techniques for the extraction of fractured abutment screws. Twenty abutment screws were randomly fractured into twenty dental implants and randomly extracted using the following removal techniques: Group A: drilling technique without irrigation (*n* = 10) (DT) and Group B: ultrasonic technique without irrigation (*n* = 10) (UT). The dental implants were submitted to a preoperative and postoperative micro-computed tomography (micro-CT) scan to obtain a Standard Tessellation Language (STL) digital file that determined the wear comparison by morphometry. Moreover, the thermographic effects generated by the DT and UT removal techniques were registered using a thermographic digital camera. Comparative analysis was performed by comparing the volumetric differences (mm^3^) between preoperative and postoperative micro-CT scans and thermographic results (°C) using the Student *t* test. The DT extracted 8/10 and the US 9/10 abutment screws. The pairwise comparison revealed statistically significant differences between the volumetric differences of postoperative and preoperative micro-CT scans of the DT (− 0.09 ± − 0.02mm^3^) and UT (− 0.93 ± − 0.32mm^3^) study groups (*p* = 0.0042); in addition, the pairwise comparison revealed statistically significant differences between the thermographic values of the DT (38.12 ± − 10.82 °C) and UT (78.52 ± 5.43 °C) study groups (*p* < 0.001). The drilling technique without irrigation provides a less removal capability, less conical internal hex implant-abutment connection damage and less thermal effect than ultrasonic technique for the extraction of fractured abutment screws; however, the ultrasonic technique resulted more effective for the extraction of fractured abutment screws.

## Background

Nowadays, dental implants are recommended as a predictable therapeutic alternative to rehabilitate partial or total edentulism [1], since they have shown high success rates with follow-ups up to 16 years [1, 2]. Moreover, dental implants have reduced the prevalence of complications associated to conventional dental-supported prostheses such as carious lesions and tooth sensitivity, in addition to maintaining the bone dimensions in the edentulous area [3]. However, dental implants are not exempt of risks and inherent biological and mechanical complications [2, 3], mainly related to the inflammatory diseases of the peri-implant tissues and the restorations of the implant-supported protheses [1, 2, 4]. Specifically, the fracture and loosening of the abutment screw of implant-supported restorations have been highlighted as the most prevalence complications [5–7]. The percentage of abutment screw fractures varies from 0 to 10.4% in studies with a 5-year follow-up [8–10], and Jung et al. reported an incidence of screw loosening of 12.7% after 5-year follow-up in implant-supported single crowns [11]. Only one study with a follow-up time of more than 20 years has been found, which shows an incidence of 29% in prosthetic fixation screw fractures [12]. Moreover, these drawbacks, can appear because of different factors as the occlusal overload., parafunctional habits, the implant connection and abutment screw design., the mechanical resistance of the restoring materials, non-passive fit and the lack or loss of adequate preload [13–16]. In addition, some authors have reported that inadequate forces generated by parafunctional habits, such as bruxism and clenching, applied to implant-supported restorations can lead to mechanical complications [2, 4, 6].

Carneiro et al. reported that special care should be taken to avoid damaging the dental implant connection, as well as the implant access channel during the fractured abutment screw removal [16]; so that the removal of a broken abutment screw is a challenging and time-consuming process due to poor visibility, especially when in internal connection type dental implants [17]. However, the removal of a broken abutment screw is also important to maintain the implant in function [18].

Some abutment screw removal systems have been developed to extract the unexpected, fractured abutment screws inside the dental implant connection, including fork-shaped instrument (FragmentFork; Dentsply Sirona) [19], a screw extraction kit (AbutmentScrewRetrieval Kit; Nobel Biocare) [20] and an implant maintenance kit (Service Kit; Straumann USA) [21]; however, there is no standardized technique to remove fractured abutment screws from the dental implants, so there are numerous techniques and devices, such as the use of a sharp bur slightly in contact with the exposed part of the screw that allows derotation of the screw [22], the use of cotton that vibrate with ultrasound [23]. Imam et al. used stainless-steel probe and instrument attached to a handpiece at low speed to remove fractured abutment screws in the apical portion of the implant but incorporates the use of ultrasonic instrumentation if this is unsuccessful [24]. Sim et al. performed a hole in the center of the screw to insert an H-file and extract the fractured abutment screw [25]. Other newer techniques have been proposed using a custom screwdriver made from a hypodermic needle [26] and Yi et al. fixes the implant-supported prosthesis with a shorter second screw, screwing it down to the fractured fragment that has not been extracted [27]. Nergiz et al. chips the fragment for removal, using a kit consisting of drill bits, 2 drill guides, and 6 hand-tapping instruments [28]. However, the conventional method in which a probe and ultrasound are used is efficient as well as economical, therefore, it is a good method for the extraction of fractured abutment screws, and this is supported by the statistical data found in the different studies that have a 73.3% extraction success with this method [29].

Moreover, the ease of removing the fractured abutment screw depends on the fracture level, since the fracture of the abutment screws occur frequently at the junction of the screw head and or at the junction where the threaded section begins [16, 17]. However, if the fractured abutment screw is not possible to be removed, the dental implant must be removed, what may increase the cost, time, and morbidity if a new dental implant needs to be placed [16]; however, Kim et al. suggested that clinically, fractured abutment screws can be replaced with shorter abutment screws without removing the remnant piece of the broken screw [30]. However, the scientific literature has not yet provided any efficient and predictable extraction protocol for the removal of abutment screw fragments from inside the dental implants, there are only a few studies on the management of these fractures, and there is also no scientific evidence to allow the clinician to choose one method or another for its effectiveness or ease of handling [29, 31], and there is no report related to the thermographic effect transferred to the peri-implant tissues during the removal fractured abutment screws neither the volumetric consequences at the implant-abutment connection after removing fractured abutment screws.

Additionally, the heat generated during the removal procedures of the fractured abutment screw can irreversibly affect to the survival of the peri-implant tissues and hence influence to the dental implant osseointegration. Kniha et al. stated that a thermal threshold between 47 and 55 °C might cause bone necrosis [32]; however, Trisi et al. reported that the exposition of bone tissues to temperatures up to 60 °C for one minute does not affect the osseointegration process [33]. Moreover, Sener et al. also highlighted the importance of irrigation to prevent temperature increasement during osteotomy site preparation [34] and Albrektsson et al. reported that external irrigation with saline at 25 °C rarely results in temperatures above the critical temperature [35].

The aim of this work was to analyze and compare the removal capability, conical internal hex implant-abutment connection damage and thermographic effect using ultrasonic and drilling techniques for the extraction of fractured abutment screws, with a null hypothesis (H_0_) stating that there will be no difference between the removal capability, conical internal hex implant-abutment connection damage and thermographic effect, between the ultrasonic and the drilling technique.

## Methods

### Study design

Twenty (20) abutment screws (Ref.: PXAS, BioHorizons, Birmingham, AL, USA) were randomly (Epidat 4.1, Galicia, Spain) assigned to twenty (20) dental implants (4.6 × 12 mm, Ref.: TLX4612 BioHorizons, Birmingham, AL, USA) and subsequently fractured inside the dental conical internal hex implant-abutment connection. Afterwards, the dental implants with the fractured abutment screws were randomly distributed (Epidat 4.1, Galicia, Spain) into the following removal techniques: Group A: drilling technique without irrigation (*n* = 10) (Neo Biotech, Seoul, Korea) (DT) and Group B: ultrasonic technique without irrigation (*n* = 10) (ProUltra^®^, Dentsply Maillefer^®^, Ballaigues, Switzerland) (UT). The randomized controlled experimental trial was performed at the Dental Centre of Innovation and Advanced Specialties at the Alfonso X El Sabio University (Madrid, Spain) between November 2021 and April 2022. The sample size was determined using a power effect of 87.2 (anything above 80 was deemed acceptable). Twenty abutment screws were included in the study in order to ensure a power effect of 80.00% for detecting statistically significant differences. The null hypothesis H_0_: μ_1_ = μ_2_ was evaluated using the bilateral Student’s *t*-test of two independent samples, with a significance level of 5.00%.

Previous studies have used irrigation during the removal procedures necessary to extract the fractured abutment screws; however, Meisberger et al. analyzed the temperature rise during removal of fractured abutment screws between two ultrasonic devices with and without cooling and concluded that the ultrasonic devices cause limited rise in temperature, even without coolant; therefore, we did not use cooling during the removal procedures since cooling reduces visibility [36]. In addition, guided drilling systems by sleeves (Neo Biotech, Seoul, Korea), do not have internal irrigation, but external, and the sleeve prevents prevent irrigation from being effective; therefore, irrigation do not reduce the temperature generated by drilling procedures [31].

### Experimental procedure

The twenty dental implants were introduced in an epoxy resin model (Ref.: 20–8130-128. EpoxiCure^®^, Buehler, IL, USA) before inducing the fracture of the abutment screws inside the conical internal hex implant-abutment connection.

**Fig. 1:**
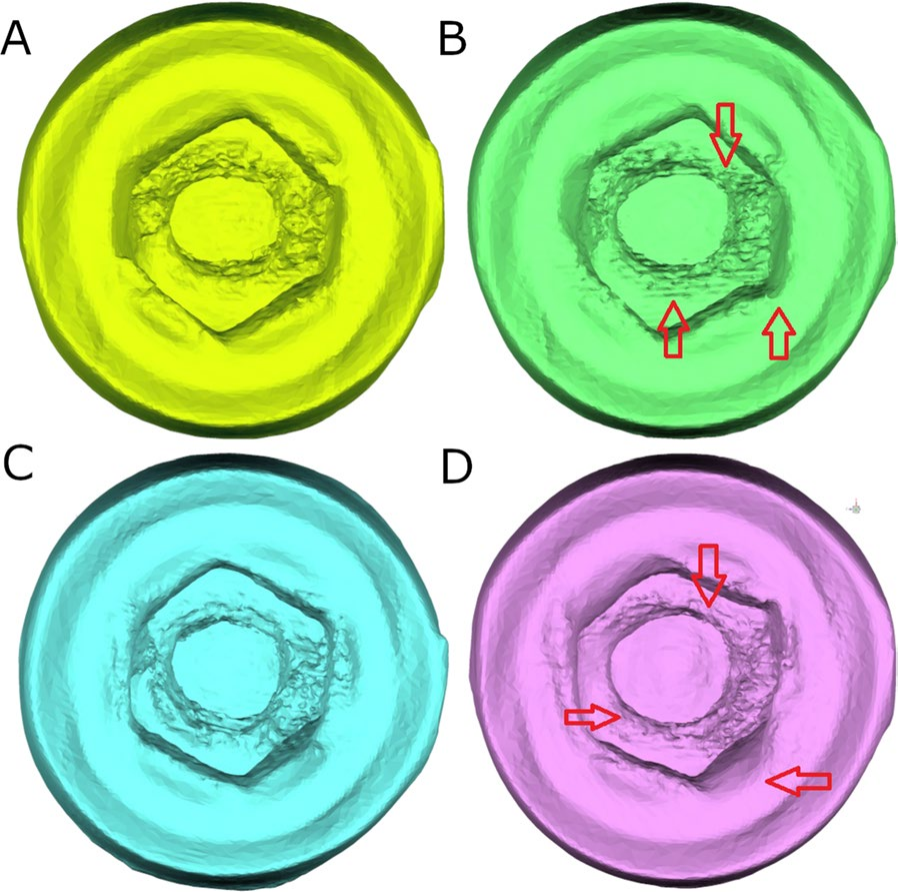
**A** Dental implant connection view of the preoperative and **B** postoperative STL digital files after removing the fractured abutment screws with drilling technique without irrigation. **C** Dental implant connection view of the preoperative and **D** postoperative STL digital files after removing the fractured abutment screws with ultrasonic technique without irrigation. The red arrows indicate the areas of wear

Then, a preoperative micro–Computed Tomography (micro-CT) scan (Super Argus MicroCT, SEDECAL, Algete, Madrid, Spain) was performed at the Molecular Imaging Laboratory, service dependent of NUCLEUS of the University of Salamanca with the following exposure parameters: 45.0 kilovolt peak, 900.0 microamperes, 720 projections, was performed to obtain accurate Standard Tessellation Language (STL) digital files of the untreated dental implants (STL1) (Fig. 1). Afterwards, the abutment screws were intentionally fractured inside the internal threads of the conical internal hex implant-abutment connection. The abutment screws were screwed at 30Ncm torque value recommended by the manufacturer and then, the abutment screws were cut 2/3 with a diamond bur (Ref.: S6881 314 012, Komet Medical, Lemgo, Germany) between the shank and threads of the abutment screws. Subsequently, the abutment screws were removed from the dental implants according to the abutment screw removal technique randomly assigned to each dental implant.

The abutment screws randomly assigned to the DT study group were removed using the Neo Screw Remover Kit II (Neo Biotech, Seoul, Korea). Firstly, the Hex 2.4 Internal Guide (Ref.: IHG24, Neo Biotech, Seoul, Korea) was placed and fixed with the SR Driver Holder (Ref.: GH00, Neo Biotech, Seoul, Korea) on the conical internal hex implant-abutment connection of the dental implants. Afterwards, the M1.6 Claw Drill was inserted (Ref.: CD16, Neo Biotech, Seoul, Korea) inside the Hex 2.4 Internal Internal Guide (Ref.: IHG24, Neo Biotech, Seoul, Korea) with the Shank Driver (Ref.: SHD00, Neo Biotech, Seoul, Korea) trying to remove manually the fractured abutment screw. However, if the fractured abutment screw could not be extracted, the 1.2 mm diameter Reverse Drill (Ref.: RCD12, Neo Biotech, Seoul, Korea) was inserted through the M2.0 Perfect Guide (Ref.: PG1220, Neo Biotech, Seoul, Korea) to perform a 1 mm-hole on the fractured surface of the fractured abutment screw, allowing the posterior use of the 1.2 mm diameter Screw Remover (Ref.: SR12, Neo Biotech, Seoul, Korea).

However, the abutment screws randomly assigned to the UT study group were removed using an ultrasonic tip (Start-X3, Dentsply SIRONA, Baillagues, Switzerland) engaged to an ultrasonic appliance (ProUltra^®^, Dentsply Maillefer^®^, Ballaigues, Switzerland) with counterclockwise circular movements and without irrigation, at 30VA power and 50 Hz frequency.

Once the abutment screws were removed or time exceeded 5 min [29], a postoperative micro-CT scan (Super Argus MicroCT, SEDECAL, Algete, Madrid, Spain) (STL2) was performed with the previously described exposure parameters (Fig. 1). Then, the STL1 and STL2 digital files were uploaded to a reverse engineering geomorphometric software (3D Geomagic Capture Wrap, 3D Systems^©^, Rock Hill, SC, USA) and an alignment procedure of the STL digital files was done with the best fit algorithm. Afterwards, the following variables were analyzed: volume assessment differences between STL1 and STL2 digital files to assess the volumetric wear. The spectrum between the alignment of STL1 and STL2 digital files was set at ± 100 µm and the tolerance at ± 10 µm. The working time necessary to remove the abutment screw from the dental implant was also recorded up to a maximum of 5 min, after this time, it was considered that the abutment screw "had not been extracted. The abutment screw removal techniques were performed by a unique operator with more than 10-years’ experience in prosthetic dentistry.

### Measurement procedure

**Fig. 2:**
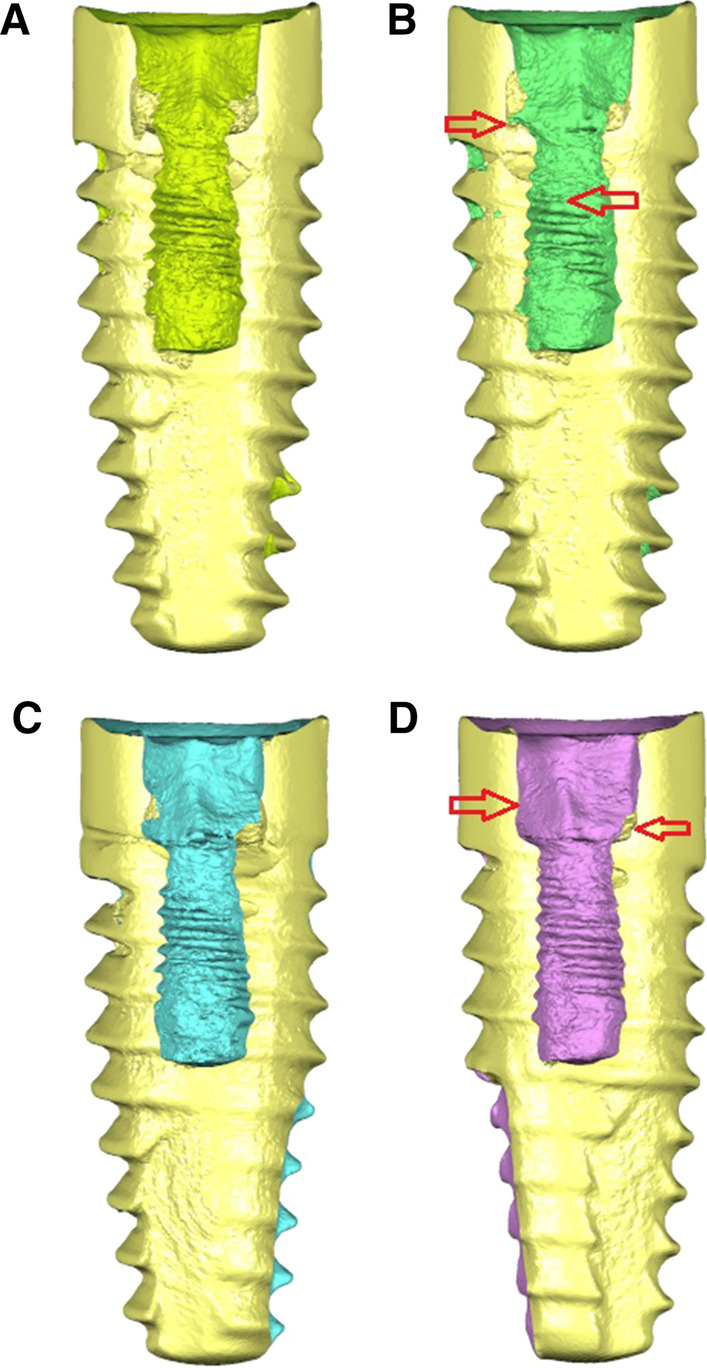
**A** Cross-section view of the preoperative and **B** postoperative STL digital files after removing the fractured abutment screws with drilling technique without irrigation. **C** Cross-section view of the preoperative and **D** postoperative STL digital files after removing the fractured abutment screws with ultrasonic technique without irrigation. The red arrows indicate the areas of wear

Area differences were also described to determine the wear of the internal threads of the conical internal hex implant-abutment connection by comparing the cross-sections after alignment of STL1 and STL2 digital files (Fig. 2).

**Fig. 3:**
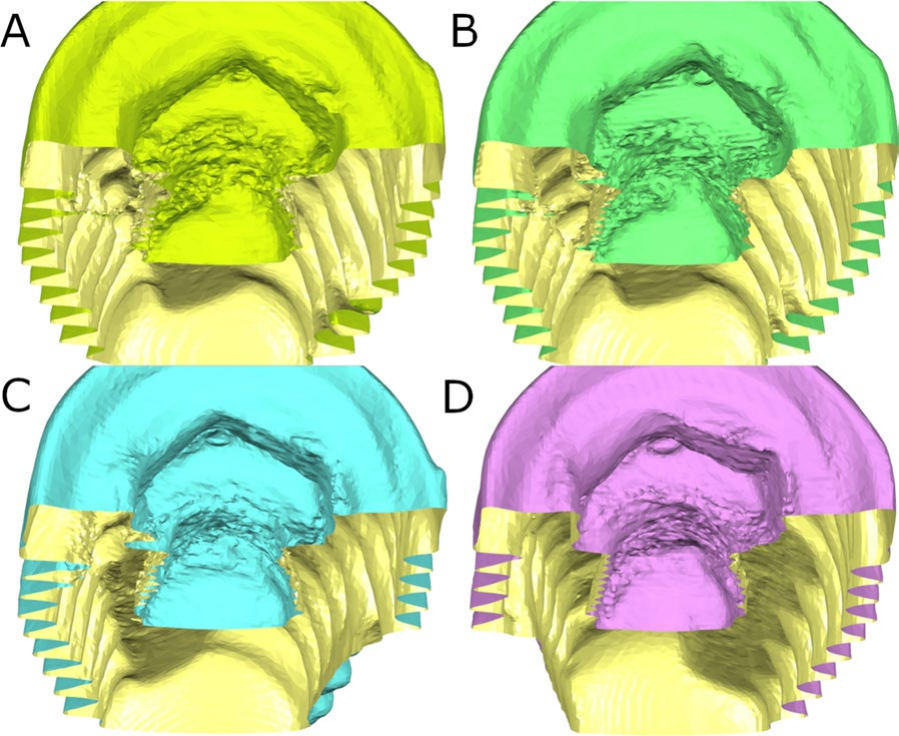
**A** Cross-section view of the preoperative and **B** postoperative STL digital files after removing the fractured abutment screws with drilling technique without irrigation. **C** Cross-section view of the preoperative and **D** postoperative STL digital files after removing the fractured abutment screws with ultrasonic technique without irrigation

Additional area measurement was performed between STL1 and STL2 digital files to determine the wear of the internal threads of the conical internal hex implant-abutment connection (Fig. 3).

### Thermal analysis

**Fig. 4:**
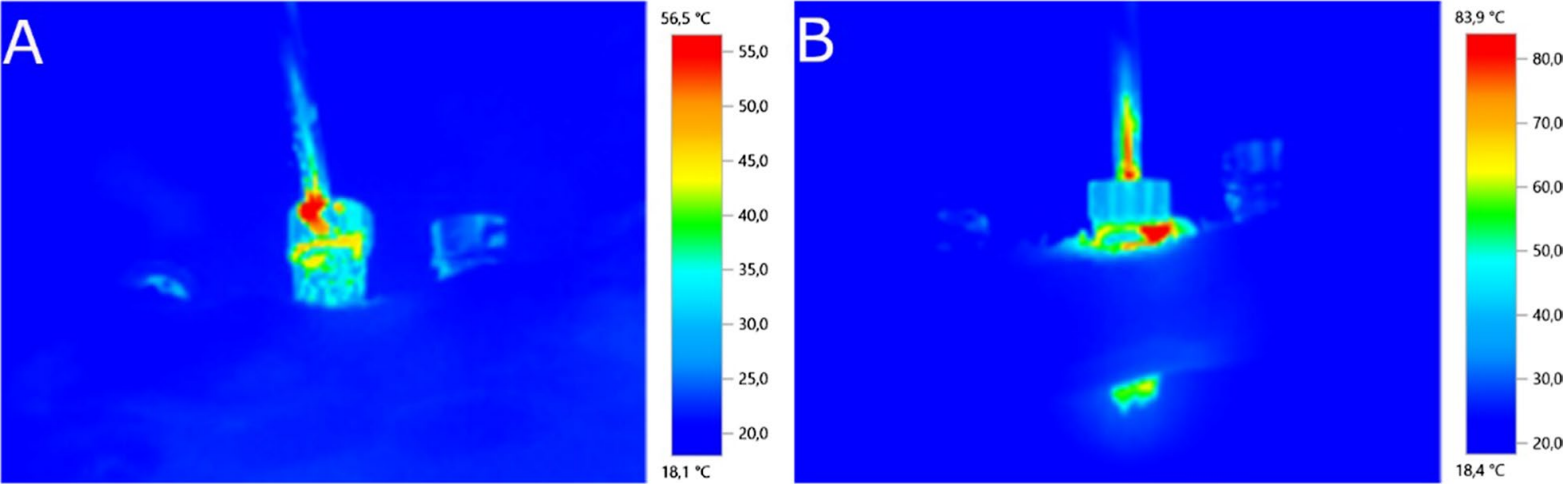
**A** The heating effect was analyzed during the removal techniques in the drilling technique without irrigation and the **B** ultrasonic technique without irrigation study groups

The heating effect generated by the abutment screw removal techniques was also analyzed by a termographic digital camera (Testo 875, Testo, Cabrils, Barcelona, Spain) placed at a distance of 2 cm [37] from the epoxy resin model surface (Ref.: 20–8130-128. EpoxiCure^®^, Buehler, IL, USA) and calibrated with a thermal range of 0–100 °C. The heating effect was analyzed during the drilling technique without irrigation (Fig. 4A) and the ultrasonic technique without irrigation (Fig. 4B).

### Statistical tests

Statistical analysis of all variables was carried out using SAS 9.4 (SAS Institute Inc., Cary, NC, USA). Descriptive statistics were expressed as means and standard deviations (SD) for quantitative variables. Comparative analysis was performed by comparing the volumetric differences (mm^3^) between postoperative and preoperative micro-CT scans and thermographic results (°C) using the Student *t* test and the Mann–Whitney non parametric test. In addition, descriptive analysis of the morphometric results (mm^3^) was performed. The statistical significance was set at *p* < 0.05.

## Results

**Fig. 5:**
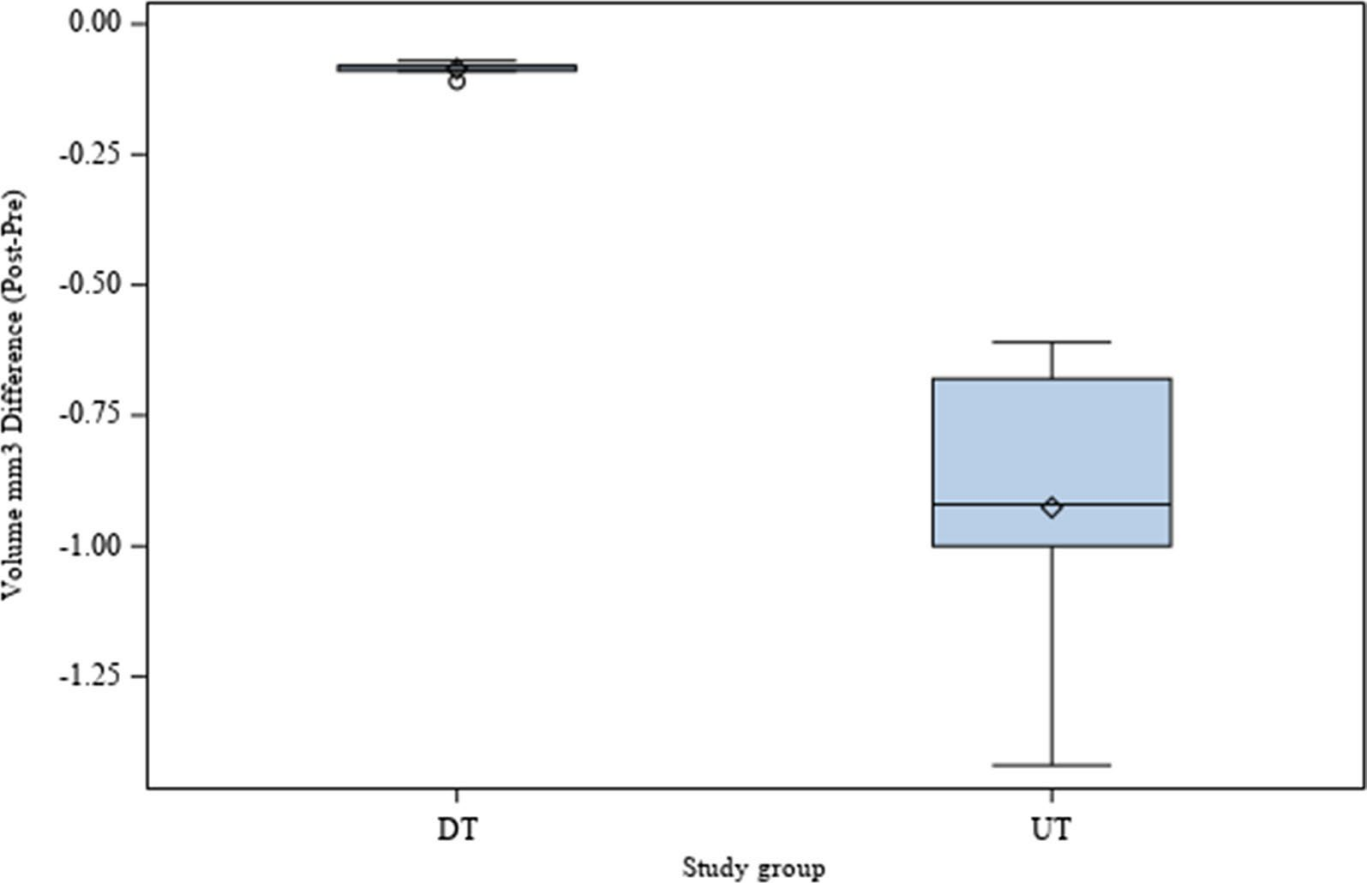
Box plots of the volumetric differences (mm^3^) between the postoperative and preoperative micro-CT scans of the DT and UT study groups. The horizontal line in each box represents median value

The means and SD values for volumetric differences (mm^3^) of postoperative and preoperative micro-CT scans of the study groups are displayed in Table 1 and Fig. 5.

**Table 1:** Descriptive statistics of the volumetric differences (mm^3^) of postoperative and preoperative micro-CT scans

Study Group	*n*	Mean	SD	Minimum	Maximum
DT	10	− 0.09 ^a^	0.02	− 0.11	− 0.07
UT	10	− 0.93 ^b^	0.32	− 1.42	− 0.61

^a, b^ different superscripts mean statistically significant differences between groups (*p* < 0.05)

The pairwise comparison revealed statistically significant differences between the volumetric differences of postoperative and preoperative microCT scans of the DT (− 0.09 ± − 0.02mm^3^) and UT (− 0.93 ± − 0.32mm^3^) study groups (*p* = 0.0042) (Fig. 5).

**Fig. 6:**
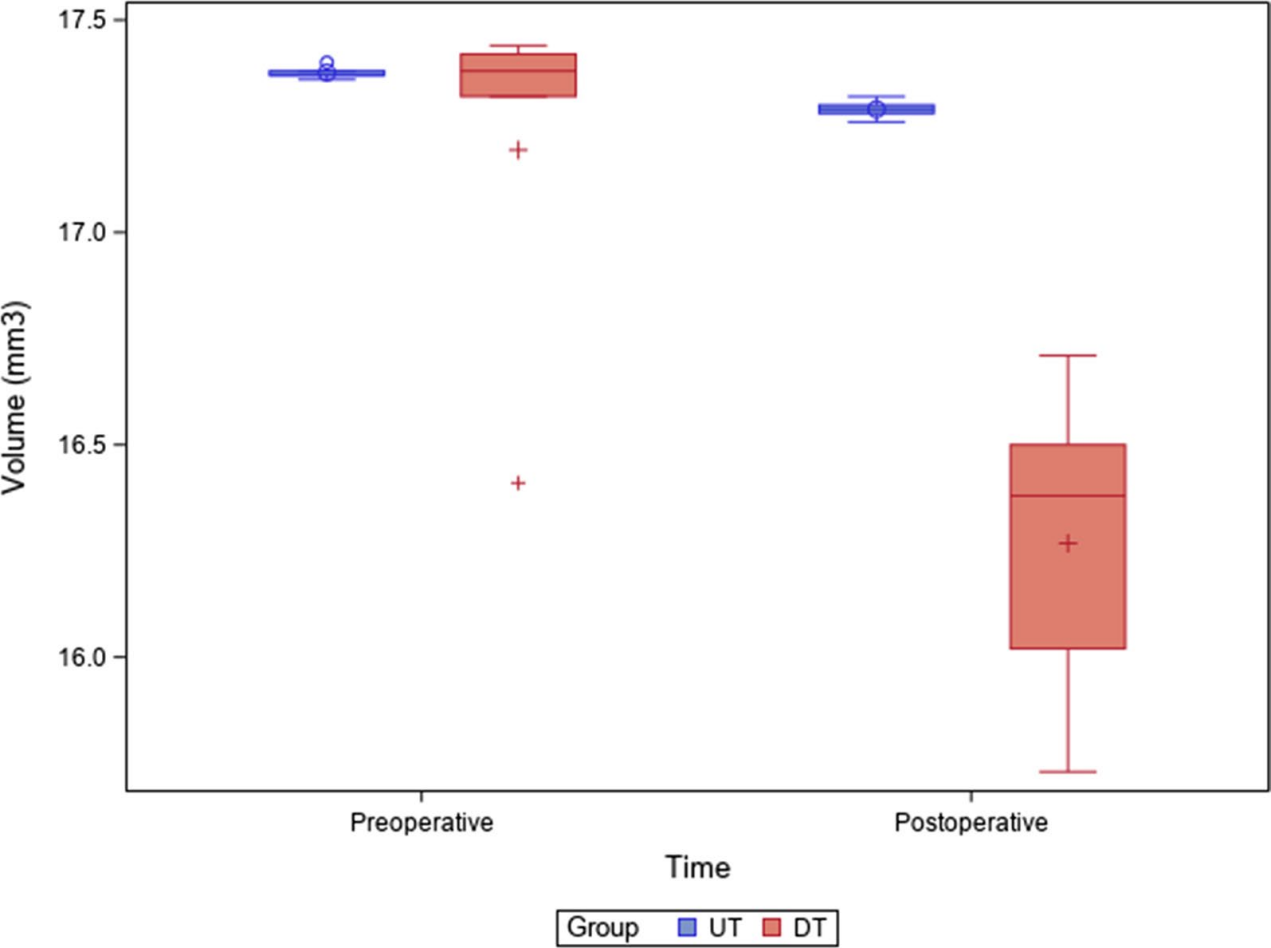
Box plots of the volumes (mm^3^) of postoperative and preoperative micro-CT scans of the DT and UT study groups. The horizontal line in each box represents median value

No statistically significant differences were shown between the preoperative volumes of the DT and UT study groups (*p* > 0.05); however, the higher postoperative wear volume associated to the UT study group showed statistically significant differences between the study groups (Fig. 6).

**Fig. 7:**
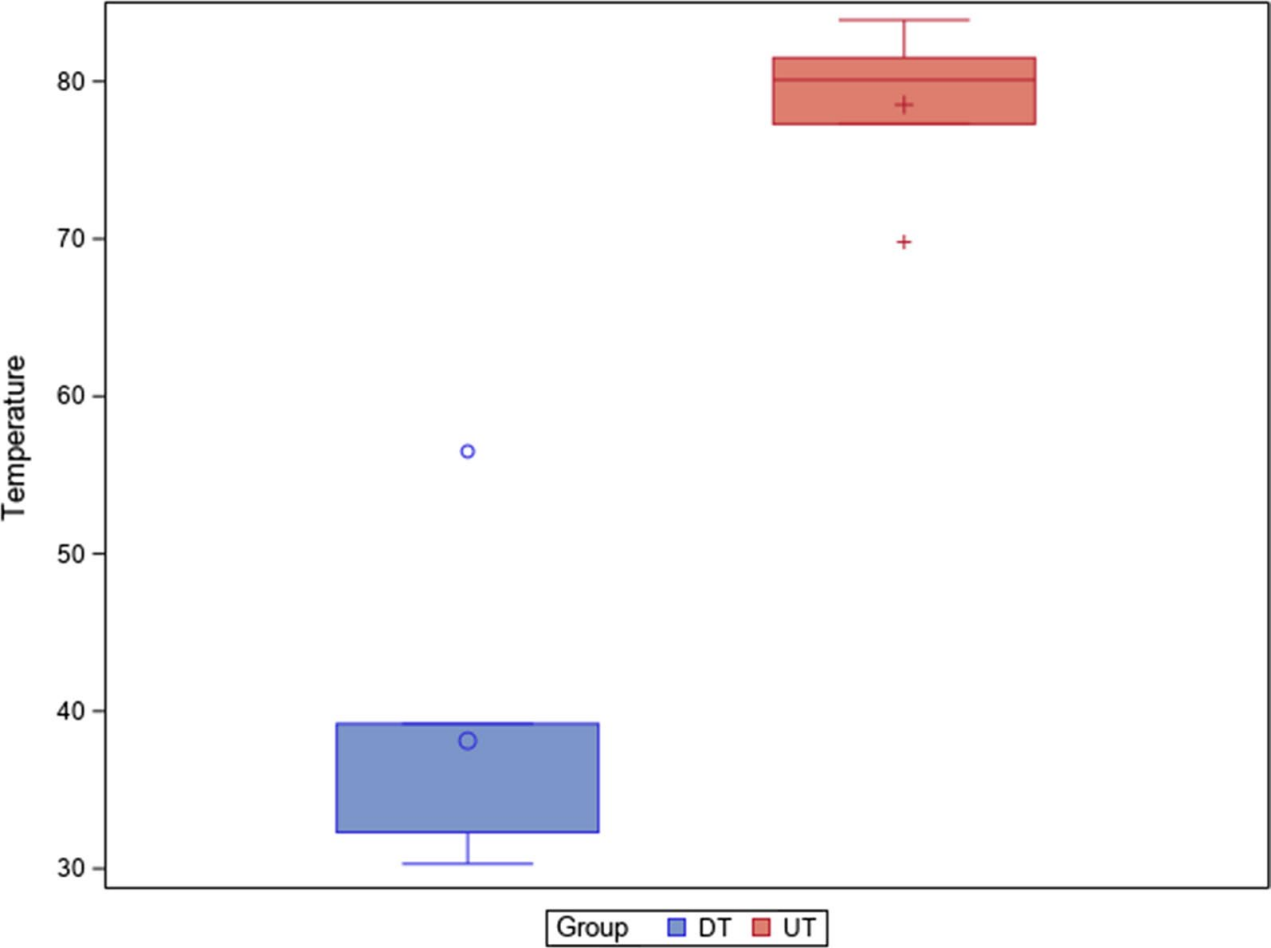
Box plots of the volumetric differences (mm^3^) between the postoperative and preoperative micro-CT scans of the DT and UT study groups. The horizontal line in each box represents median value

The means and SD values for thermographic differences (°C) between the study groups are displayed in Table 2 and Fig. 7.

**Table 2:** Descriptive statistics of the thermographic differences (°C) between the study groups

Study Group	*n*	Mean	SD	Minimum	Maximum
DT	10	38.12 ^a^	10.82	30.30	56.50
UT	10	78.52 ^b^	5.43	69.80	83.90

^a, b^ different superscripts mean statistically significant differences between groups (*p* < 0.05)

The pairwise comparison revealed statistically significant differences between the thermographic values of the DT (38.12 ± − 10.82 °C) and UT (78.52 ± 5.43 °C) study groups (*p* < 0.001) (Fig. 7).

Finally, the drilling technique without irrigation managed to remove eight out of ten fractured abutment screws; however, the ultrasonic technique without irrigation managed to remove nine out of ten fractured abutment screws, within the established 5 min working time. After this time, it was considered that the abutment screw "had not been extracted.

## Discussion

The results obtained in the present study rejected the null hypothesis (H_0_) that states that there would be no difference between the removal capability, conical internal hex implant-abutment connection damage and thermal effect, between the ultrasonic and the drilling technique.

This article showed that the drilling technique without irrigation consisting of partially perforating the fractured abutment screw to allow unscrewing, was slightly less effective than the ultrasonic technique without irrigation; although the later led to a higher conical internal hex implant-abutment connection and a greater thermal effect, suitable to be transferred to the peri-implant tissues. However, the drilling technique without irrigation is not easy to transfer to a clinical setting since it requires the placement of inserts in the dental implant that requires a large mouth opening, specially in posterior location dental implants.

However, the ultrasonic technique has been widely analyzed within the area of endodontics relating to the removal of fractured posts [38, 39] and NiTi alloy endodontic rotary files that can provide useful information in the field of implantology. In the literature, it is stated that the type of ultrasound device, ultrasound power setting, tip geometry, dentin thickness, and canal configuration can affect the temperature rise during post removal [40]. However, ultrasounds may lead to a temperature rise which can also impact on the surrounding periodontal tissues [41]. Sauk et al. reported that the exposition of the periodontal ligament cells to a temperature of 43 °C resulted in protein desnaturation leading to ankylosis and bone resorption [42, 43] Moreover, other investigations have shown morphologically bone tissue damage above 47 °C [44] and permanent bone tissue damage between 56 and 60 °C with the inactivation of alkaline phosphatase at 56 °C, considered the critical temperature of denaturation [42]. Specifically, Eriksson and Albrektsson found that the application of 47 °C of temperature for 1 min conditioned the survival of bone tissue in rabbits. This result suggests that the critical temperature for bone tissue damage is time dependent under 47 °C. Moreover, Dominici et al. reported that the endodontic post removal technique using ultrasonic vibration without irrigation also exceeded 47 °C of temperature after 15 s [42, 44, 45]. In addition, Satterthwaite et al. showed that most of extracted teeth (46/60) submitted to endodontic post removal exceeded 47 °C of temperature after 5 min of ultrasonic vibration [42, 44]. Additionally, Huttula et al. concluded that the ultrasonic vibration increases the temperature over 47C which irreversible bone damage may occur, even though a heat sink was used [40, 42, 46, 47]. Furthermore, reported that the application of a ultrasonic thechnique without irrigation for endodontic post removal for 4 min generated enough heat at the root surface to potentially affect the adjacent teeth [48] However, Gooty et al. recommended the use of an ultrasonic scaler to successfully remove a fractured abutment screw by creating a 1 mm depth hole in the occlusal surface of the fractured abutment screw by a round bur, followed by placing the tip of the scaler in this slot to further unscrew the fractured abutment screw [49]. Additionally, the vibrating tip of a piezoelectric ultrasonic scaler can be run on the top surface of the fractured screw with gentle reverse torque to drive it out of the screw hole [50]. Chen and Cho suggested the use of a Hu-Friedy TU17/23 double-ended explorer to rotate the fragment counterclockwise. If this technique fails, a stiffer hand scaler was used to engage the fractured surface. If the fragment is still not retrievable, a dental restorative adhesive backing (True Grip; Clinician's Choice) was used to hook the top of the fragment and rotate it clockwise initially followed by counterclockwise rotation [51]. However, Huang et al. reported that the removal success of the fractured abutment screw is directly dependent on the initial situation of the fractured abutment screw [56]. When conservative approaches to remove fractured abutment screws have failed, some authors have recommended the use of commercial remover kits. The IMZ Twin Plus Repair Set K 3.3 (Dentsply Friadent, Mannheim, Germany) was used successfully to remove a fractured abutment screw. [56] The kit consists of three drilling guides, four drills, a conical instrument to recover the fragment and a tapping instrument. After roughening the fragment at its center, the 1.3 and 1.9 mm twist drills are used to drill the fragment at its center, turning clockwise. Drill guides are attached to the top of the implants to protect the internal aspect of the implants from the drills. Subsequently, the conical retriever instrument is inserted into the drilled hole and the fragment is unscrewed.

Murat et al. reported that different removal techniques have been developed for retrieving abutment screw fragments inside the dental implant connection [52]. Moreover, previous studies reported that the nano axial movements generated during prosthetic loading could lead to the fracture of the abutment screw [53–55]. According to Huang et al., internal connections, anti- rotational and conical designs may increase the resistance to abutment screw loosening; however, cantilevers may also increase the risk of abutment screw fractures [56]. Moreover, Byrne et al. and Park et al. suggested that abutment screws coated with a diamond–like carbon (DLC) surface reduce the friction of internal threats, providing higher preloads and consequently, reducing the risk of loosening the abutment screws [57, 58].

The accurate analysis (tolerance at ± 10 µm) of the volumetric wear of the dental conical internal hex implant-abutment connection after the extraction of fractured abutment screws by means of the morphometric technique validates this measurement procedure for future studies in which it is necessary to analyze volumetric changes. However, the accurate of this measurement technique is associated with the resolution of the STL digital files to be compared, therefore it is advisable to use digital files with a high density of tesellas such as those provided by micro-CT. Nevertheless, further clinical research is needed to determine the clinical relevance of the implant-abutment damage and heat generation and on the prognosis of dental implants.

## Conclusions

In conclusion, within the limitations of this study, our results showed that the drilling technique without irrigation provides a less removal capability, less conical internal hex implant-abutment connection damage and less thermal effect than ultrasonic technique for the extraction of fractured abutment screws; however, the ultrasonic technique resulted more effective for the extraction of fractured abutment screws.

## Data Availability

The datasets used and/or analysed during the current study are available from the corresponding author on reasonable request.
